# Identification of B-cell maturation antigen (BCMA)-targeting peptides and selective drug delivery to multiple myeloma using peptide-targeted DM1 prodrug-loaded nanoparticles

**DOI:** 10.1039/d6na00398b

**Published:** 2026-10-09

**Authors:** Gyoyeon Hwang, Raeleen H. Sobetski, Doga E. Karakus, Emily K. Bromley, Basar Bilgicer

**Affiliations:** a Department of Chemical and Biomolecular Engineering, University of Notre Dame Notre Dame IN USA bbilgicer@nd.edu; b Department of Chemistry and Biochemistry, University of Notre Dame Notre Dame IN USA; c Mike and Josie Harper Cancer Research Institute, University of Notre Dame Notre Dame IN USA; d Berthiaume Institute for Precision Health, University of Notre Dame Notre Dame IN USA

## Abstract

Selective delivery of anticancer therapeutics to tumor cells, without affecting healthy tissues, is crucial for effective cancer treatment. Recently, targeted therapies that exploit B-cell maturation antigen (BCMA), highly overexpressed on multiple myeloma (MM) cells, have emerged, demonstrating promising therapeutic efficacy. However, high-affinity ligands targeting BCMA may bind to low-level BCMA expression on normal cells, posing risks of off-target toxicity. To address this issue, we designed BCMA-targeting peptides (BCMApep) and identified BCMApep1 with a low affinity. BCMApep1 was multivalently conjugated on a liposomal nanoparticle surface (TNP^BCMApep1^), achieving selective and enhanced uptake by MM cells *via* the avidity effect. Furthermore, we loaded a mertansine (DM1) prodrug within the BCMA-targeted nanoparticles (TNP^BCMApep1^[DM1]), which demonstrated significantly superior cytotoxicity and apoptosis in MM cells upon pulse dosing compared to DM1 prodrug-loaded non-targeted nanoparticles (NP[DM1]). Overall, our study establishes a BCMA-targeting, peptide-guided nanoparticle platform for selective delivery of the DM1 prodrug to MM cells. The enhanced efficacy of TNP^BCMApep1^[DM1] highlights its potential as a safe and effective therapeutic strategy for multiple myeloma.

## Abstract 2

Identification of a low-affinity BCMA-targeting peptide and its multivalent display on liposomes enable selective uptake in multiple myeloma cells and enhance the therapeutic efficacy of a DM1 prodrug while potentially reducing off-target toxicity.

## Graphical abstract

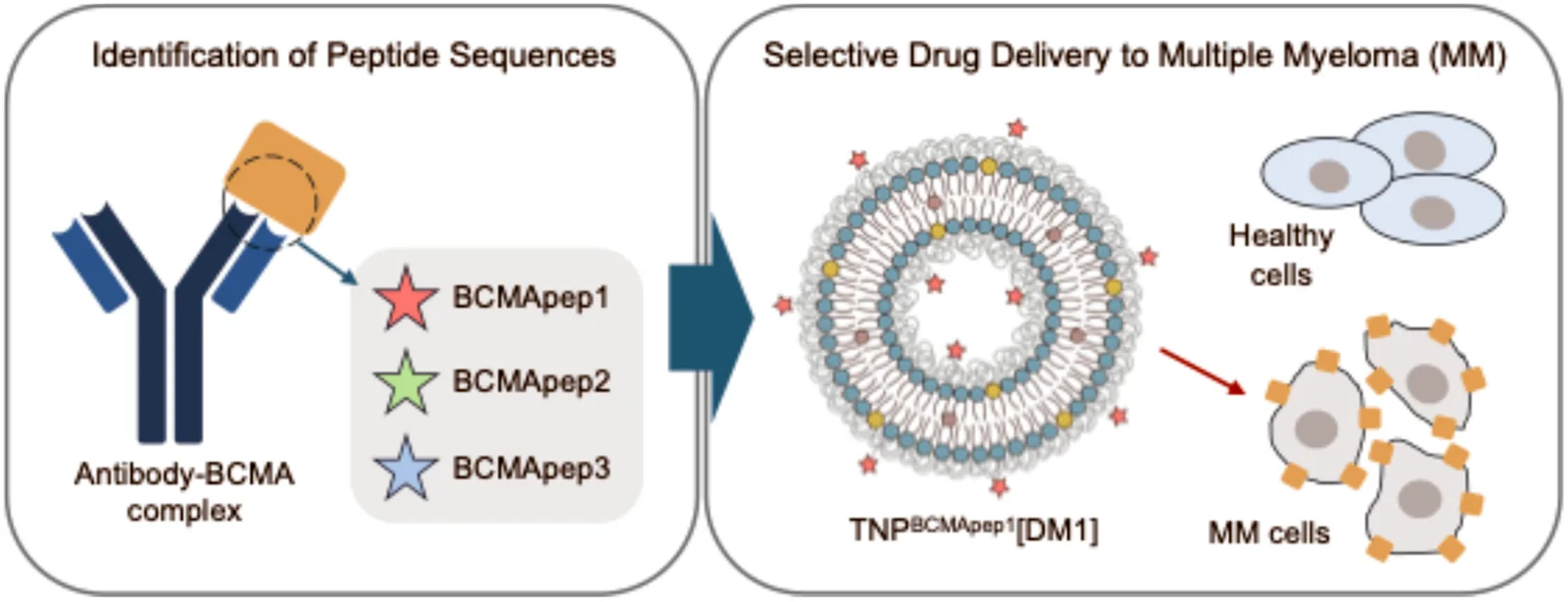

## Introduction

Multiple myeloma (MM) is the second most common hematologic malignancy and accounts for approximately 2% of all cancers in the United States and worldwide.^1,2^ MM is a plasma cell malignancy characterized by focal solid lesions that infiltrate the bone marrow, leading to lytic bone lesions, defective hematopoiesis, and renal failure.^3^ Despite advancements in chemotherapy and immunotherapy, MM remains largely incurable due to disease relapse and resistance to current treatment regimens.^4,5^ One of the key challenges in MM therapy is the lack of a specific targeting agent that can effectively distinguish malignant plasma cells from normal cells, thereby minimizing off-target effects and enhancing therapeutic efficacy.^5^ In this context, the development of targeted therapies holds significant promise for improving treatment outcomes in MM.

Active targeted therapies have transformed the treatment landscape of MM by enabling precise delivery of therapeutics to malignant plasma cells, thereby improving efficacy and reducing systemic toxicity.^5,6^ These approaches include monoclonal antibodies (mAb), antibody–drug conjugates (ADCs), chimeric antigen receptor (CAR) T cell therapy, and bispecific T cell engager (BiTE).^6,7^ Despite advances with targeted therapies, these therapies use targeting molecules with high monovalent affinity like antibodies, which are too strong to achieve selectivity between healthy and cancerous cells that both display the target receptor, that cause insufficient selectivity and remain a major limitation.

Instead of relying on high monovalent affinity moieties, low monovalent affinity ligands such as carbohydrates, peptides, and small molecules have been applied to target their receptors, where multivalent display compensates for the weak affinity and restores high binding.^8–11^ Our lab has engineered a platform to use the weak-to-moderate affinity of peptides to selectively target various cells by loading the targeting peptide multivalently on the surface of liposomal nanoparticles.^12–15^ By using this approach, we can engineer a system that binds with high avidity to cancer cells that overexpress the targeted receptor while interacting weakly with healthy cells that have low receptor expression. This approach accomplishes selectivity only for the cancer cells using the engineered targeted nanoparticles (TNPs) with the right design parameters. For MM, our lab has been investigating the targeting of receptors including very late antigen-4 (VLA-4), leukocyte Peyer's patch adhesion molecule-1 (LPAM1), CD38, and CD138, demonstrating their potential for targeted MM therapies.^12,13,16,17^ Nevertheless, these receptors are simultaneously expressed by healthy cells; additionally some receptors are not overexpressed in cancer cells for all patients, which could potentially hinder their clinical success. Therefore, there is a need for improving our arsenal of novel peptides that target critical receptors in MM to achieve better selectivity hence better outcomes.

B-Cell maturation antigen (BCMA) is a vital target for MM targeted therapy, as recent research has shown that targeting BCMA can lead to significant improvements, which have shown potent and durable responses in heavily pretreated patients.^18–23^ BCMA is a member of the tumor necrosis factor receptor superfamily and plays a significant role during plasma cell differentiation and survival through interaction with its ligands, B cell activating factor (BAFF) and a proliferation-inducing ligand (APRIL).^24^ BCMA is exclusively expressed on the cell membrane of late-stage B cells and plasma cells and regulates differentiation and survival of plasma cells. BCMA is selectively expressed by differentiated plasma cells but not by B lymphocytes, hematopoietic stem cells, or non-hematopoietic tissue.^25^ In addition, the overexpression and activation of BCMA are associated with progression of MM in preclinical models and humans.^26,27^ We hypothesize that the identification of BCMA-targeting peptides (BCMApep) and their multivalent presentation on liposomal nanoparticle platforms could improve therapeutic specificity and minimize unintended off-target effects.

Here, we conducted an investigation to identify the BCMApep with low-moderate affinity from the crystal structure of antigen–antibody complexes. We then investigated the selective binding of BCMApep towards MM cell lines and targeted uptake of liposomal nanoparticles displaying the peptide multivalently by MM cells. Competitive binding assays provided robust validation of the specificity of BCMApep on the nanoparticles to the BCMA receptor. Furthermore, we conducted comprehensive analyses of the cytotoxic potential exhibited by mertansine (DM1) prodrug-loaded targeted nanoparticles against MM cells. Collectively, the data presented herein establish the therapeutic potential of BCMA-targeted nanoparticles, highlighting their significant promise as a targeted therapeutic modality in the selective treatment of MM.

## Materials and methods

### Materials

TentaGel S RAM resin was purchased from Chem-Impex (Wood Dale, IL). *N*,*N*-Dimethylformamide (DMF), piperidine, *N*,*N*,*N*′,*N*′-tetramethyl-*O*-(1*H*-benzotriazol-1-yl)uronium hexafluorophosphate (HBTU), *N*,*N*-diisopropylethylamine (DIEA), trifluoroacetic acid (TFA), triisopropylsilane (TIS), isopropyl alcohol (IPA), cholesterol, chloroform, phosphate buffered saline (PBS), bovine serum albumin (BSA), Fmoc-amino acids, 5(6)-carboxyfluorescein, and 3,3-dioctadecyloxacarbocyanine perchlorate (DiO) were purchased from MilliporeSigma (Burlington, MA). 1,2-Distearoyl-*sn*-glycero-3-phosphocholine (DSPC) and 1,2-distearoyl-*sn*-glycero-3-phosphoethanolamine-*N*-[methoxy(polyethylene glycol)-2000] (ammonium salt) (PEG2000-DSPE) were purchased from Avanti Research (Birmingham, AL). Alexa Fluor® 647 anti-human BCMA antibody and 7-aminoactinomycin D (7-AAD) were purchased from BioLegend (San Diego, CA). Mertansine (DM1) was purchased from MedChemExpress (Monmouth Junction, NJ). Cell counting kit-8 (CCK-8) reagent was purchased from Dojindo (Rockville, MD). Acetonitrile (ACN) was purchased from VWR (Radnor, PA). l-Glutamine (200 mM), penicillin–streptomycin (10 000 U mL^−1^), 2-mercaptoethanol, and Annexin V-Alexa Fluor® 488 were purchased from Thermo Fisher Scientific (Waltham, MA). Fmoc-PEG_2_-OH and Fmoc-PEG_8_-OH were purchased from Vector Laboratories (Newark, CA). Trypsin–EDTA and RPMI-1640 were purchased from Corning (Glendale, AZ). Fetal bovine serum (FBS) was purchased from Cytiva (Marlborough, MA). DSPE-maleimide was purchased from Nanosoft Polymers (Winston-Salem, NC).

### Synthesis of peptides and peptide–lipid conjugates

Peptide–lipid conjugates, soluble peptides, and fluorescein isothiocyanate (FITC)–peptide conjugates were synthesized *via* solid phase peptide synthesis (SPPS) using Fmoc chemistry. The Fmoc-protected resin-bound peptide chain was deprotected with 20% piperidine in DMF for 3 min, repeated three times. The C-terminal carboxyl group of the amino acid, linker, or lipid was activated with HBTU and DIEA in DMF prior to coupling to the resin. Coupling was monitored using the Kaiser test. The completed product was cleaved from the resin using 95%/2.5%/2.5% TFA/TIS/water, twice for 45 min each. Purification was performed by reversed-phase HPLC (RP-HPLC) using an Agilent 1200 HPLC system (Agilent Technologies, Santa Clara, CA). Soluble peptides and FITC–peptide conjugates were purified using a Zorbax C18 column with an ACN gradient, while peptide–lipid conjugates were purified using a Zorbax C3 column with an IPA gradient. Elution was monitored using absorbance in the 200–600 nm range. Purified products were characterized using a Bruker microOTOF-QII mass spectrometer (Bruker, Billerica, MA). The chemical structures and corresponding mass spectrometry data of all synthesized molecules are presented in Fig. S1 and Table S1.

### Synthesis of the DM1 prodrug

The DM1 prodrug was synthesized as previously described.^28^ DM1 and DSPE-maleimide were reacted at a 1 : 1.2 molar ratio in DMF at room temperature overnight. Purification was performed by RP-HPLC using a Zorbax C3 column with an IPA gradient and subsequently characterized using a Bruker micrOTOF-QII mass spectrometer. The chemical structure and mass spectrometry data of the DM1 prodrug are presented in Fig. S2 and Table S1.

### Preparation of liposomal nanoparticles

A mixture of bulk lipids (DSPC), PEG2000–lipid conjugates (PEG2000-DSPE), peptide–lipid conjugates, DiO dye or DM1 prodrug, and cholesterol was prepared in chloroform and dried under a stream of nitrogen gas to form a thin lipid film. The film was hydrated with PBS (pH 7.4) at 68 °C under rotation and then extruded at the same temperature through a 50 nm polycarbonate filter membrane using an Avanti Polar Lipids extruder (Avanti Research, Birmingham, AL). Liposomes were formulated using the following molar ratio relative to total lipid content: (95 − *x* − *y*) : 5 : *x* : *y* : 5 for DSPC : PEG2000-DSPE : peptide–lipid conjugates : DiO or DM1 prodrug : cholesterol. We used *x* = 0, 0.1, 0.2, 0.25, 0.5, 0.75, 1, or 2, and *y* = 0.4 for DiO dye and *y* = 5 for the DM1 prodrug.

### Characterization of nanoparticles

The size distribution of the prepared liposomal nanoparticles was measured using a NanoBrook Omni particle size analyzer (Brookhaven Instruments, Nashua, NH). The loading efficiency of peptide–lipid conjugates or the DM1 prodrug incorporated into the liposomes was evaluated by analyzing the area under the curve (AUC) at 220 nm or 280 nm in absorbance chromatograms obtained from an Agilent 1200 HPLC system. Liposomes were purified using a liposome extruder purification (LEP) with a 30 nm polycarbonate filter membrane, following a previously developed method, prior to HPLC analysis.^29^ Zeta potential of prepared nanoparticles was measured at pH 7.4 using the Smoluchowski approximation on a NanoBrook Omni particle size analyzer.

### Cell culture

NCI-H929, MM.1S, MM.1R, U266, IM-9, RPMI-8226, Raji, and MOLT-4 cell lines were obtained from the American Type Culture Collection (Rockville, MD). AMO1 cells were kindly provided by Dr Kenneth C. Anderson (Dana-Farber Cancer Institute, MA). NCI-H929 cells were cultured in RPMI-1640 medium supplemented with 20% FBS, 2 mM l-Glutamine, 100 U mL^−1^ penicillin, 100 µg mL^−1^ streptomycin, and 55 µM 2-mercaptoethanol. MM.1S, MM.1R, U266, IM-9, RPMI-8226, Raji, and MOLT-4 cells were cultured in RPMI-1640 medium supplemented with 10% FBS, 2 mM l-glutamine, 100 U mL^−1^ penicillin, and 100 µg mL^−1^ streptomycin.

### Receptor profiling and cell surface binding assay

For analysis of surface receptor expression, 1 × 10^5^ cells per well were incubated with Alexa Fluor® 647 anti-human BCMA antibody in blocking buffer (1.5% BSA in PBS) for 30 min on ice. Cells were then washed twice with PBS and analyzed using a Guava EasyCyte 8HT flow cytometer (MilliporeSigma, Burlington, MA). For the monovalent peptide binding assay, 1 × 10^5^ cells per well were plated in 24-well plates and pre-incubated with the blocking buffer (1.5% BSA in PBS) for 45 min on ice. Cells were then incubated with increasing concentration of FITC-labeled peptide in blocking buffer for 1 h on ice. For the competitive binding inhibition assay, cells were first blocked and then incubated with excess soluble free peptide (100 µM) for 30 min on ice. Subsequently, cells were treated with 100 µM lipid concentration of DiO-labeled liposomes at varying peptide densities and incubated for 1 h on ice. After incubation, all cells were analyzed using the Guava EasyCyte 8HT flow cytometer.

### Cellular uptake assay

1 × 10^5^ cells per well in 24-well plates were incubated with DiO-labeled liposomes at a lipid concentration of 100 µM with varying peptide densities for 3 h at 37 °C. After incubation, cells were washed with PBS and treated with 0.25% Trypsin–EDTA solution to remove surface-bound liposomes. Following an additional PBS wash, cells were analyzed using the Guava EasyCyte 8HT flow cytometer.

### Cell cytotoxicity assay

2 × 10^4^ cells per well were plated in 96-well plates and incubated overnight at 37 °C. For continuous dosing, cells were treated with DM1, DM1 prodrug, NP[DM1], or TNP^BCMApep1^[DM1] for 48 h at 37 °C. For pulse dosing, cells were incubated with each formulation for 1, 3, or 6 h, washed with PBS, and then maintained in a fresh medium for the remaining time to complete a 48-h incubation. Cell viability was assessed using the CCK-8 reagent according to the manufacturer's instructions.

### Apoptosis assay

2 × 10^4^ cells per well were plated in 96-well plates and incubated overnight at 37 °C. Cells were then treated with the DM1 prodrug, NP[DM1], or TNP^BCMApep1^[DM1] for 1, 3, or 6 h, washed with PBS, and subsequently maintained in a fresh culture medium for the remaining time to complete a 48-h incubation. Apoptosis was assessed using Annexin V-Alexa Fluor® 488/7-AAD staining. Stained cells were analyzed on a Guava EasyCyte 8HT flow cytometer, and data were quantified using FlowJo software (BD Biosciences, Franklin Lakes, NJ).

### Statistical analyses

All experiments were performed in triplicate unless otherwise specified. Data represent mean ± SD with statistical significance levels: ***, *P* < 0.001; **, *P* < 0.01; *, *P* < 0.05. *P*-Values were assessed by an unpaired *t*-test and obtained using GraphPad Prism 11 (GraphPad Software, Boston, MA).

## Results and discussion

### Identification of low-affinity BCMApep

To identify prospective BCMApep sequences, we examined two crystal structures of two anti-human BCMA antibodies as complexes with BCMA, J22.9-xi (PDB ID: 4ZFO) and UniAb (PDB ID: 6J7W) (Fig. 1A). J22.9-xi is an antibody that blocks the binding of the native BCMA ligands APRIL and BAFF. With its high affinity (*K*_d_ ∼ 54 pM), the antibody effectively depletes MM cells *in vitro* and *in vivo* and prolongs tumor-free survival in a xenograft mouse model.^30^ We analyzed the complementarity determining regions (CDRs) of the Fab fragment. From these analyses, we selected the linear sequence of seven amino acids FSASLRF (residues 49–55, termed BCMApep1), which contained numerous interactions with BCMA at the binding interface. UniAb is a heavy chain-only antibody (HCAb) that retains high affinity for BCMA (*K*_d_ ∼ 300 pM) while existing as the smallest active antibody fragment.^31^ From UniAb, we also recognized the linear sequence PEWVSGIRGS (residues 45–54, termed BCMApep2) for its binding potential to BCMA. We additionally engineered a variant of this sequence, PEWVSVSRGS (termed BCMApep3), where two amino acids in BCMApep2 were modified to improve binding. Glycine (residue 51), although oriented toward BCMA, is a non-interacting residue; hence it was replaced with valine to introduce an additional hydrophobic contact with BCMA. Additionally, isoleucine (residue 52), a hydrophobic residue positioned away from the binding interface, was substituted with serine, a moderately hydrophilic residue. The introduction of serine favors interaction with solvent, reducing potential unintended interactions with BCMA while improving solubility of the peptide.

**Fig. 1:**
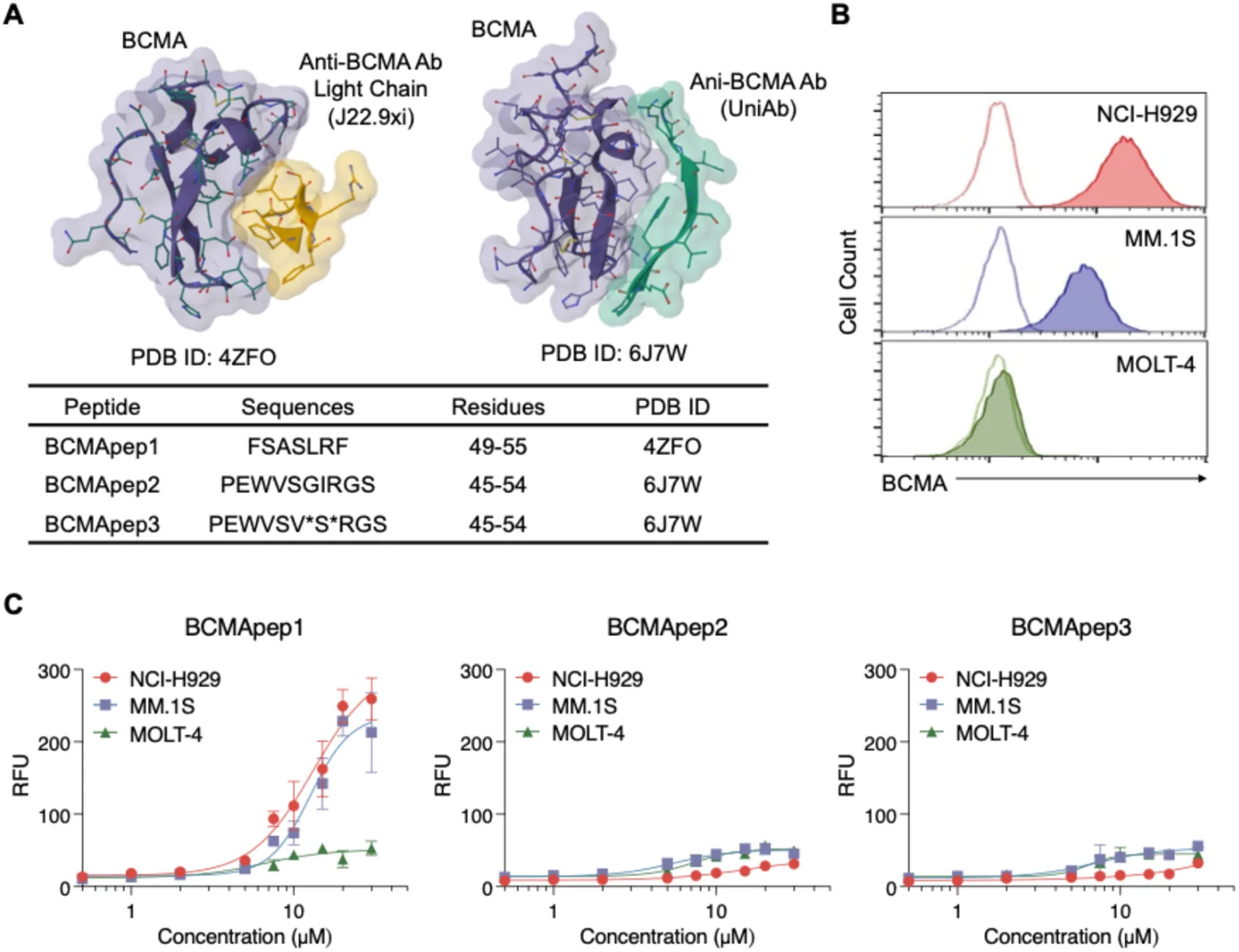
Identification of BCMA-antagonist peptides (BCMApep). (A) Crystal structures of BCMA in complex with the peptide sequence in the anti-BCMA antibodies J22-xi (PBD ID: 4ZFO) and UniAb (PBD ID: 6J7W). The asterisk (*) indicates the amino acids in BCMApep3 that were modified from BCMApep2. (B) BCMA expression on human multiple myeloma (MM) cell lines NCI-H929 and MM.1S and the human T-cell leukemia cell line MOLT-4. Cells were immunostained with Alexa Fluor 647-labeled anti-BCMA antibodies, and expression was compared with that of untreated controls. (C) Cellular binding of FITC-labeled BCMApep1, BCMApep2, and BCMApep3. The binding of antibodies and peptides was determined by flow cytometry. All experiments were performed in triplicate, and data represent mean ± SD.

Before evaluating these peptides, we confirmed the cell-surface expression levels of BCMA on MM cell lines by antibody staining. BCMA was readily expressed on human MM cell lines, NCI-H929 and MM.1S, while it was minimal on the MOLT-4 human T lymphocyte leukemia cell line (Fig. 1B). We next performed cellular binding assays to validate the binding affinity of the peptides to BCMA on the cells. FITC labeled monovalent BCMApep1, BCMApep2, and BCMApep3 were synthesized and their binding to NCI-H929, MM.1S, and MOLT-4 cells was detected using flow cytometry. BCMApep1 displayed apparent binding to BCMA on NCI-H929 and MM.1S cells as the concentration of BCMApep1 increased, but showed negligible binding to MOLT-4 due to a lack of BCMA receptor expression on these cells. The *K*_d_ of BCMApep1 to BCMA was determined to be 13 µM (weak-to-moderate affinity). Meanwhile, BCMApep2 and BCMApep3 did not exhibit any appreciable binding to any of the cell lines (Fig. 1C). While the sequences were derived from the CDRs of the aforementioned Fab fragments, linear peptides typically suffer from entropic penalties upon target due to their flexibility.^32^ Despite this limitation, BCMApep1 demonstrated considerable binding, likely due to intramolecular π–π stacking interaction between two phenylalanine residues located at the termini of BCMApep1, which may be stabilizing its conformation and thereby reducing the entropic cost of binding for a linear peptide. This structural stabilization potentially contributes to the enhanced receptor affinity observed for BCMApep1. The results indicate BCMApep1 to be the winning peptide candidate among the ones tested here that will have the highest chance for selectively targeting the BCMA-expressing MM cells.

### Preparation and characterization of TNP^BCMApep^

We first prepared BCMA-targeted nanoparticles (TNP^BCMApep^) by incorporating BCMApep–lipid conjugates in liposomal nanoparticle membranes for their multivalent presentation. Our lab has developed a liposomal nanoparticle preparation method that accomplishes manufacturing of TNPs with precise and consistent composition while eliminating batch to batch variability. This is accomplished by incorporating pre-synthesized and purified peptide–lipid conjugates into the liposomes during formulation at the exact desired stoichiometric ratios (Fig. 2A). Using pure peptide–lipid conjugates during liposome assembly, instead of first assembling liposomal particles and then functionalizing their surfaces using surface-conjugation techniques, results in reproducibility since starting with pre-purified components yields pure particles and eliminates the need for a follow up purification which is almost impossible with targeted particles. The peptide–lipid conjugate was designed as previously described.^12,14,15^ It consists of BCMApep as targeting ligands, an EG_2_ spacer, lysine residues to improve the partitioning of the BCMApep into the aqueous phase, an EG_8_ linker to extend the BCMApep above the PEG cloud, a tryptophan residue to determine concentration, and two lipid tails for strong anchoring to the lipid bilayer of liposomes (Fig. 2B). After extruding non-targeted nanoparticles (NPs, 0% peptide density) and TNP^BCMApep^ with varying peptide densities (0.1%, 0.25%, 0.5%, 0.75%, and 1%) of BCMApep1 (TNP^BCMApep1^), BCMApep2 (TNP^BCMApep2^), and BCMApep3 (TNP^BCMApep3^), the size distribution of particles was determined using dynamic light scattering (DLS). All TNP formulations had a size of ∼75 nm with homogeneous particle populations and narrow size polydispersity (Fig. 2C and Table S2). Additionally, TNP^BCMApep^ was analyzed to confirm whether BCMApep were loaded at a desired ratio in the liposomes. TNP^BCMApep1^, TNP^BCMApep2^, and TNP^BCMApep3^ were prepared at varying densities of BCMApep1, BCMApep2, and BCMApep3, respectively and subjected to liposome extruder purification (LEP) to remove the peptide–lipid conjugates that were not incorporated.^29^ The quantities of incorporated BCMApep–lipid conjugates in particles were measured using RP-HPLC. In all TNP^BCMApep^ formulations, the peptide loading efficiency was greater than 97% following LEP (Fig. S3A). This high loading efficiency indicates minimal peptide loss during the liposome extrusion process. Furthermore, the trendline obtained from the RP-HPLC analysis after LEP exhibited strong linear correlations between the measured peptide amounts and the desired stoichiometric ratios (Fig. S3B). These results confirm that our method of TNP preparation can be precisely adjusted to the desired targeting molecule density with high size uniformity.

**Fig. 2:**
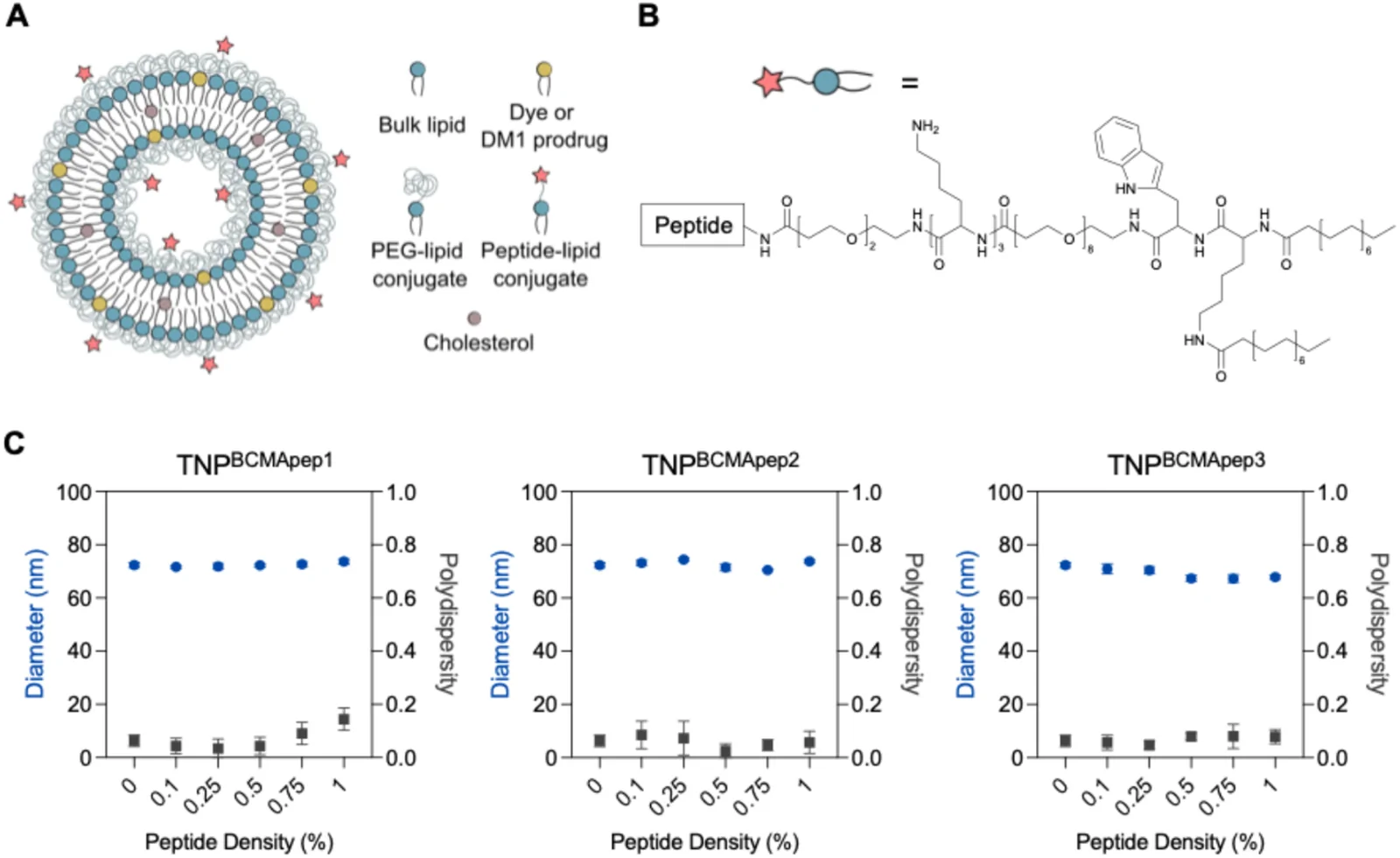
Characterization of BCMA-targeted nanoparticles (TNP^BCMApep^). (A) Schematic illustration of targeted nanoparticles. (B) Structure of the peptide–lipid conjugate. (C) Size measurement of non-targeted nanoparticles (NPs; 0% peptide density) and targeted nanoparticles with varying peptide densities (0.1%, 0.25%, 0.5%, 0.75%, and 1%) of BCMApep1 (TNP^BCMApep1^), BCMApep2 (TNP^BCMApep2^), and BCMApep3 (TNP^BCMApep3^). The measurement was performed using dynamic light scattering (DLS) and data represent mean ± SD (*n* = 5).

### Cellular uptake evaluation of TNP^BCMApep^ formulations

To verify the selective particle endocytosis performed only by BCMA-expressing cells, we performed cellular uptake assays with NPs and TNP^BCMApep^ at varying densities (0.1–1%) of BCMApep1, BCMApep2, and BCMApep3. NCI-H929, MM.1S, and MOLT-4 cells were treated with these nanoparticles for 3 h and the particle uptake was evaluated *via* flow cytometry. TNP^BCMApep1^ showed superior cellular uptake in NCI-H929 and MM.1S cells across all peptide loading conditions, with uptake increasing as peptide density increased. Notably, minimal uptake was observed in MOLT-4 cells, confirming strong selectivity for MM cells. For TNP^BCMApep2^ and TNP^BCMApep3^, cellular uptake in MM cells also increased with higher peptide density, but there was a notable difference in the peptide-density threshold required for significant uptake compared to TNP^BCMApep1^. While TNP^BCMApep1^ showed significant particle uptake in MM cells even at 0.1% peptide density, TNP^BCMApep2^ and TNP^BCMApep3^ exhibited notable uptake starting from 0.75% and 0.5% peptide density, respectively (Fig. 3A). To quantitatively analyze the selective targeting of MM cells, we calculated the fold uptake-enhancement by MM cell lines (NCI-H929 and MM.1S) over MOLT-4. For TNP^BCMApep1^, the fold uptake-enhancement increased with increasing peptide density, reaching a maximum of 150-fold in NCI-H929 and 100-fold in MM.1S at 0.75% peptide density. Nevertheless, at 1% peptide density, the fold uptake-enhancement was less pronounced, possibly due to non-specific binding to MOLT-4 cells, which reduced selectivity. In contrast, TNP^BCMApep2^ exhibited a 50-fold uptake enhancement at 1% peptide density and TNP^BCMApep3^ reached a plateau at 40-fold in NCI-H929 and 25-fold in MM.1S from 0.75% peptide density (Fig. 3B). The lower fold enhancement observed for TNP^BCMApep2^ and TNP^BCMApep3^ compared to TNP^BCMApep1^ is due to their affinity as monovalent peptides shown in Fig. 1C. Taken together, these findings suggest that multivalent presentation of BCMApep1 on the liposomal nanoparticles facilitates more efficient and selective uptake in MM cells at lower peptide densities compared to BCMApep2 and BCMApep3. In addition, it is worth nothing that excessive peptide valency can increase avidity to the point of inducing off-target effects. Therefore, optimizing peptide density on the nanoparticle surface is critical to achieving a balance between high avidity and target selectivity.

**Fig. 3:**
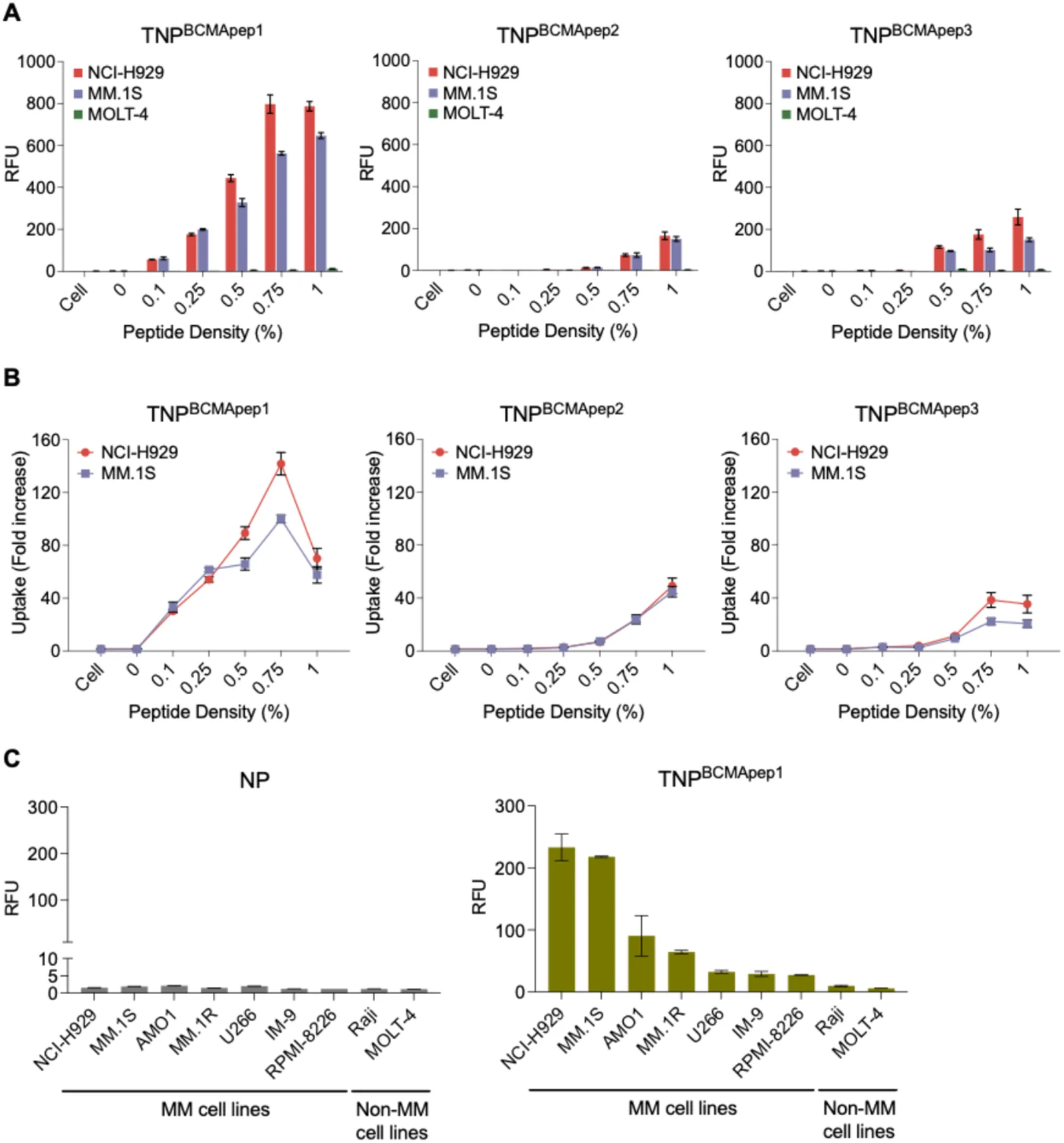
Cellular uptake of NPs and TNP^BCMApep^. (A) Uptake of NPs, TNP^BCMApep1^, TNP^BCMApep2^, and TNP^BCMApep3^ with varying peptide densities (0.1%, 0.25%, 0.5%, 0.75%, and 1%) by human MM cell lines (NCI-H929 and MM.1S) and a human T-cell leukemia cell line (MOLT-4). Cells were incubated with DiO-labeled nanoparticle formulations for 3 h, and uptake was measured by flow cytometry. (B) Fold increase in particle uptake by NCI-H929 cells and MM.1S cells relative to MOLT-4 cells, calculated from the relative fluorescence unit (RFU) obtained in panel A, respectively. (C) Uptake of NPs and TNP^BCMApep1^ (0.5% BCMApep1 density) by seven human MM cell lines (NCI-H929, MM.1S, AMO1, MM.1R, U266, IM-9, and RPMI-8226), a human Burkitt's lymphoma cell line (Raji), and MOLT-4. Cells were incubated for 3 h with each formulation, and uptake was quantified by flow cytometry. All experiments were performed in triplicate, and data represent mean ± SD.

To further validate the selective targeting capability of TNP^BCMApep1^, we assessed particle uptake in additional cell lines. Seven different human MM cell lines (NCI-H929, MM.1S, AMO1, MM.1R, U266, IM-9, and RPMI-8226) and two human non-MM cell lines (Raji and MOLT-4) were incubated with NPs and TNP^BCMApep1^ for 3 h. Quantitative analysis revealed that internalization of NPs by any of the tested cell lines with no BCMA expression was negligible. In contrast, TNP^BCMApep1^ exhibited significant uptake in all MM cell lines (Fig. 3C). These results further reinforce that TNP^BCMApep1^ was selectively taken up by MM cells with high BCMA expression, while exhibiting minimal uptake in non-MM cells with limited expression, confirming potential significance of BCMA targeting in MM-specific drug delivery. Based on these results, we chose TNP^BCMApep1^ as the lead candidate for the continuation of the study.

### Evaluation of BCMApep1 specificity

To examine the specificity of BCMApep1 binding, competitive binding inhibition assay was performed by co-incubating soluble BCMApep1. NCI-H929 and MM.1S cells were first incubated in an excessive concentration of BCMApep1, followed by the addition of fluorescent dye-labeled TNP^BCMApep1^ formulations with various BCMApep1 densities (0.5%, 1%, and 2%). Following further incubation, TNP^BCMApep1^ binding to cells for each formulation was assessed using flow cytometry. In the presence of free BCMApep1, the binding of TNP^BCMApep1^ was drastically reduced in both NCI-H929 and MM.1S cells for all formulations (Fig. 4A). Free BCMApep1 competitively inhibited the binding of TNP^BCMApep1^ at 0.5% and 1% BCMApep1 density by ∼80% on both cell lines and TNP^BCMApep1^ at 2% BCMApep1 density on NCI-H929 and MM.1S by ∼70% and 50%, respectively (Fig. 4B). The particles loaded with a higher density (2%) of BCMApep1 showed less inhibition of binding compared to those loaded with lower BCMApep1 densities (0.5% and 1%). This observation can be explained due to the increase in binding avidity as a result of increased peptide density. The results demonstrate that BCMApep1 is highly specific to the BCMA receptor.

**Fig. 4:**
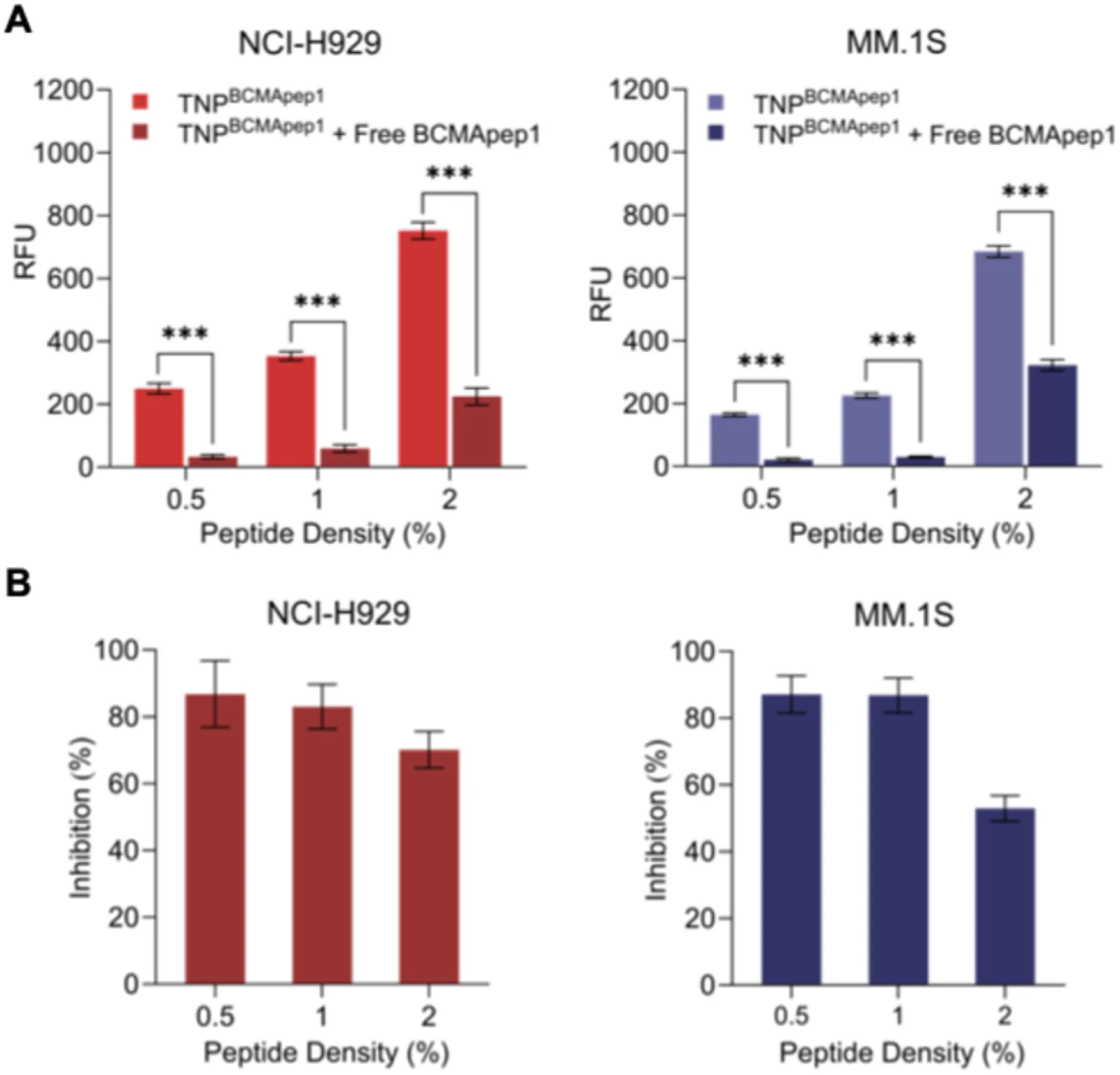
Assessment of BCMApep1 specificity. (A) Competitive inhibition of TNP^BCMApep1^ binding by free BCMApep1. NCI-H929 and MM.1S cells were incubated with excessive free BCMApep1 for 30 min, followed by the addition of DiO-labeled TNP^BCMApep1^ (0.5%, 1%, and 2% BCMApep1 density) for 1 h. TNP^BCMApep1^ binding was then measured by flow cytometry. Statistical significance represents comparison of cellular binding in groups between TNP^BCMApep1^ and TNP^BCMApep1^ with free BCMApep1. *P*-Values were calculated using an unpaired *t*-test. ***, *P* < 0.001. (B) Percent inhibition of TNP^BCMApep1^ binding to NCI-H929 and MM.1S cells, calculated from the RFU obtained from data in panel (A). All experiments were performed in triplicate, and data represent mean ± SD.

### Cytotoxicity of DM1 prodrug-loaded TNP^BCMApep1^ (TNP^BCMApep1^[DM1])

In a previous publication, we reported how a prodrug version of mertansine (DM1), which is a tubulin inhibitor chemotherapeutic, was developed to enhance its therapeutic index and pharmacokinetic properties. The DM1 prodrug loaded CD138-targeted nanoparticle formulation was shown to reduce DM1's off-target toxicity during MM treatment.^28^ To evaluate the MM cell toxicity of liposomal nanoparticles with the DM1 prodrug payload, we first prepared DM1 prodrug-loaded NPs (NP[DM1]) and DM1 prodrug-loaded TNP^BCMApep1^ (TNP^BCMApep1^[DM1]). DM1 prodrug loading did not markedly alter the particle size or zeta potential (Table S2 and Fig. S4). Moreover, incorporation of BCMApep1 did not compromise the DM1 loading efficiency (Fig. S5). Next, NCI-H929 cells were incubated with varying concentration of free DM1 molecules, the free DM1 prodrug, NP[DM1], and TNP^BCMApep1^[DM1] for 24, 48, or 72 h. Free DM1, the free DM1 prodrug, NP[DM1], and TNP^BCMApep1^[DM1] showed efficacy against MM cells in 24 h of continuous exposure, with IC_50_ values of ∼20, ∼500, ∼100, and ∼80 nM, respectively (Fig. 5A). The free DM1 prodrug showed lower therapeutic potential than both nanoparticle formulations and free DM1, resulting from its stable linkage before its inactive form is transformed into its active form inside the cells. The difference in the IC_50_ values between the free DM1 prodrug and both particle formulations is likely due to variations in the intracellular conversion kinetics of the prodrug into active DM1. Nanoparticle formulations may promote endocytosis-mediated internalization of the DM1 prodrug, while the free DM1 prodrug exhibits limited uptake. As the incubation time increased, the cytotoxic effects of nanoparticle formulations became comparable to those of free DM1, as IC_50_ values of free DM1, NP[DM1], and TNP^BCMApep1^[DM1] at 48 h were determined to be ∼10 nM, ∼40 nM, and ∼30 nM, respectively and IC_50_ values of free DM1, NP[DM1], and TNP^BCMApep1^[DM1] at 72 h were determined to be ∼10 nM, ∼30 nM, and ∼20 nM, respectively. In contrast, the DM1 prodrug alone did not show cytotoxicity comparable to that of free DM1, as indicated by its significantly higher IC_50_ values of ∼250 nM and ∼200 nM at 48 and 72 h, respectively. These findings suggest that while NP[DM1] and TNP^BCMApep1^[DM1] can achieve similar cytotoxic effects as free DM1 over extended incubation periods, the DM1 prodrug alone is less effective when it is not incorporated in a liposomal formulation.

**Fig. 5:**
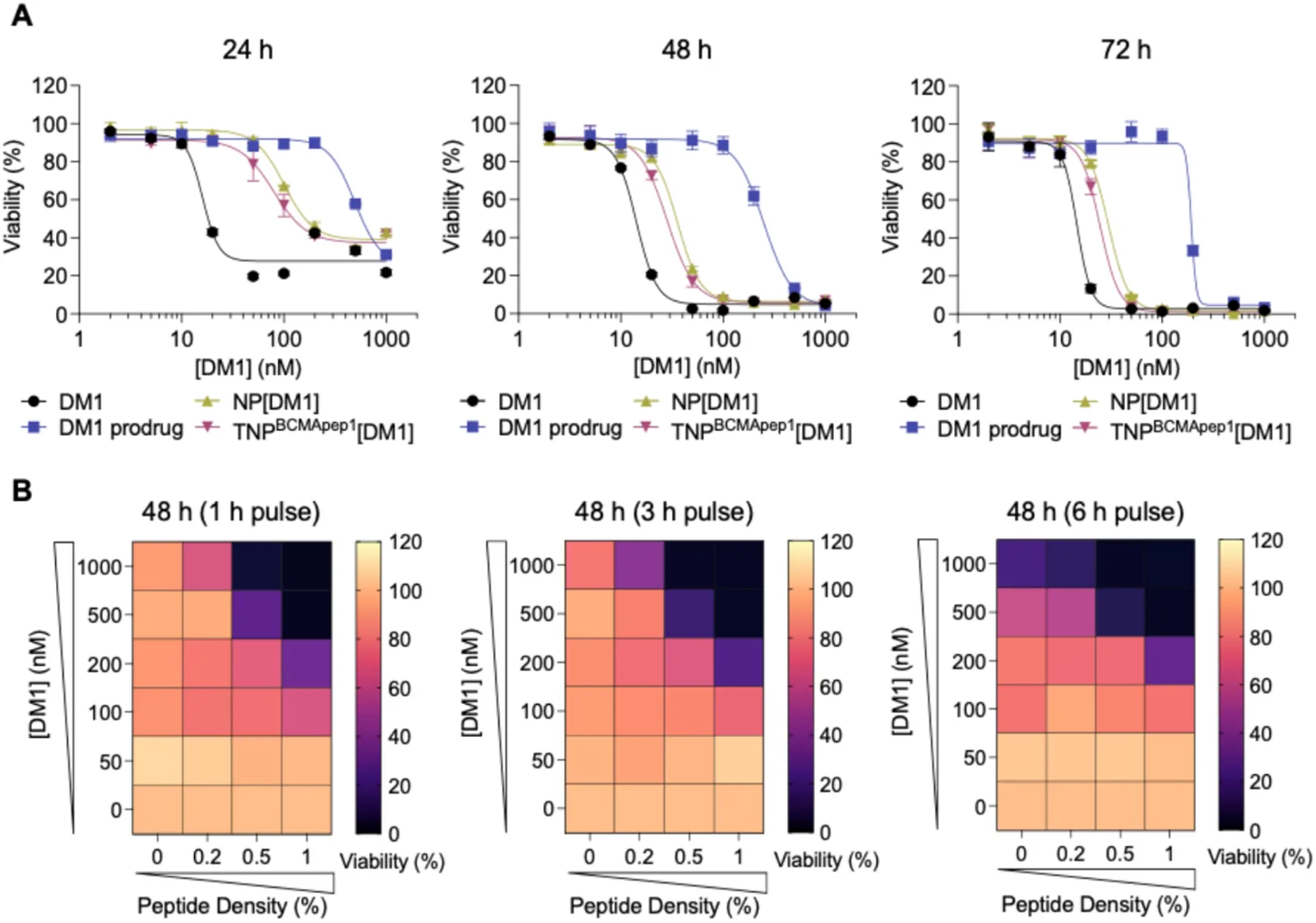
Cytotoxicity evaluation of DM1 prodrug-loaded liposomal nanoparticles. (A) Continuous exposure of NCI-H929 cells to free DM1, free DM1 prodrug, DM1 prodrug-loaded NPs (NP[DM1]), or DM1 prodrug-loaded TNP^BCMApep1^ (TNP^BCMApep1^[DM1]) for 24, 48, or 72 h. For continuous dosing, TNP^BCMApep1^[DM1] was prepared with 0.5% BCMApep1 density. All experiments were performed in triplicate, and data are presented as mean ± SD. (B) Pulse dosing approach in NCI-H929 cells using NP[DM1] (0% BCMApep1 density) and TNP^BCMApep1^[DM1] at varying BCMApep1 densities (0.2%, 0.5%, and 1%). Cells were incubated with each formulation for 1, 3, or 6 h, washed with PBS, and then maintained in fresh media for the remaining time to complete a 48 h-incubation. Data represent the mean from three independent experiments.

In physiology, nanoparticles are under constant circulation through the bloodstream rather than staying dormant next to cancer cells. To assess the impact of enhanced targeted drug delivery under more clinically relevant conditions, the efficacy of pulse dosing with NP[DM1] (0% BCMApep density) and TNP^BCMApep1^[DM1] formulations containing various densities (0.2%, 0.5%, and 1%) of BCMApep1 was evaluated. Pulse dosing consists of brief drug exposure followed by washout and incubation in a fresh medium. This approach better mimics the limited binding half-life and dynamic circulation behavior of formulations in physiological settings. NCI-H929 cells were treated with varying DM1-equivalent concentrations of NP[DM1] and TNP^BCMApep1^[DM1] under pulse dosing conditions (1, 3, or 6 h), followed by incubation in a fresh medium for the remaining time to complete 48 h-incubation. A higher density of BCMApep1 on TNP^BCMApep1^[DM1] led to a greater reduction in MM cell viability at all pulse incubation times, particularly at DM1-equivalent concentrations exceeding 100 nM (Fig. 5B and S6). Notably, BCMApep1, on its own, did not induce any cytotoxicity within the concentration range used in the assay (Fig. S7). These results confirm that the enhanced targeting capability of TNP^BCMApep1^[DM1] contributes to their therapeutic efficacy without introducing additional cytotoxic effects from the targeting element itself. In contrast to TNP^BCMApep1^[DM1], NP[DM1], represented by the leftmost column in all heat maps, exhibited significant cell killing only at the longest pulse incubation time (6 h) and at higher DM1-equivalent concentration (≥500 nM) (Fig. 5B and S6). This result is likely due to the absence of specific targeting interactions, which limits cellular internalization during short pulse exposures. Therefore, only prolonged exposure and elevated drug concentrations increase the probability of non-specific adsorption of NP[DM1], which compensates for their inefficient cellular uptake. Based on these results, TNP^BCMApep1^[DM1] demonstrates clear therapeutic advantages over NP[DM1] by achieving robust cytotoxic efficacy under transient, physiologically relevant conditions, thereby enabling effective drug delivery at lower concentrations.

### Assessment of apoptosis in MM cells with treatment of TNP^BCMApep1^[DM1]

We next assessed apoptosis in single cells using Annexin V-Alexa Fluor 488/7-amino-actinomycin D (7-AAD) staining, after administrating the DM1 prodrug, NP[DM1], and TNP^BCMApep1^[DM1]. At 3 h of pulse dosing to NCI-H929 cells and an additional 45 h of incubation, we observed that the TNP^BCMApep1^[DM1] exhibited a higher proportion of both early apoptotic (Annexin V+/7-AAD−) and late apoptotic (Annexin V+/7-AAD+) cell populations over control, DM1 prodrug, and NP[DM1] groups (Fig. 6A and B). The relatively high early apoptosis and late apoptosis fraction show that TNP^BCMApep1^[DM1] triggers apoptotic pathways with active DM1 in the cells using increased intracellular delivery of particles and sustains its cytotoxic effects over the full observation period. By contrast, cells treated with the free DM1 prodrug or NP[DM1] showed comparatively lower levels of Annexin V positivity, which suggests that the active DM1 concentration for inducing the apoptotic pathway could not be reached due to reduced intracellular uptake within the pulse dosing period. This result highlights the importance of the targeting moiety in enhancing active drug accumulation within the MM cells in accordance with the cytotoxicity assay results shown in Fig. 5B. Taken together, these findings confirm that TNP^BCMApep1^[DM1] delivery not only accelerates internalization of particles with the drug but also maintains effective intracellular concentration of active DM1, leading to an increase in overall apoptotic cell death.

**Fig. 6:**
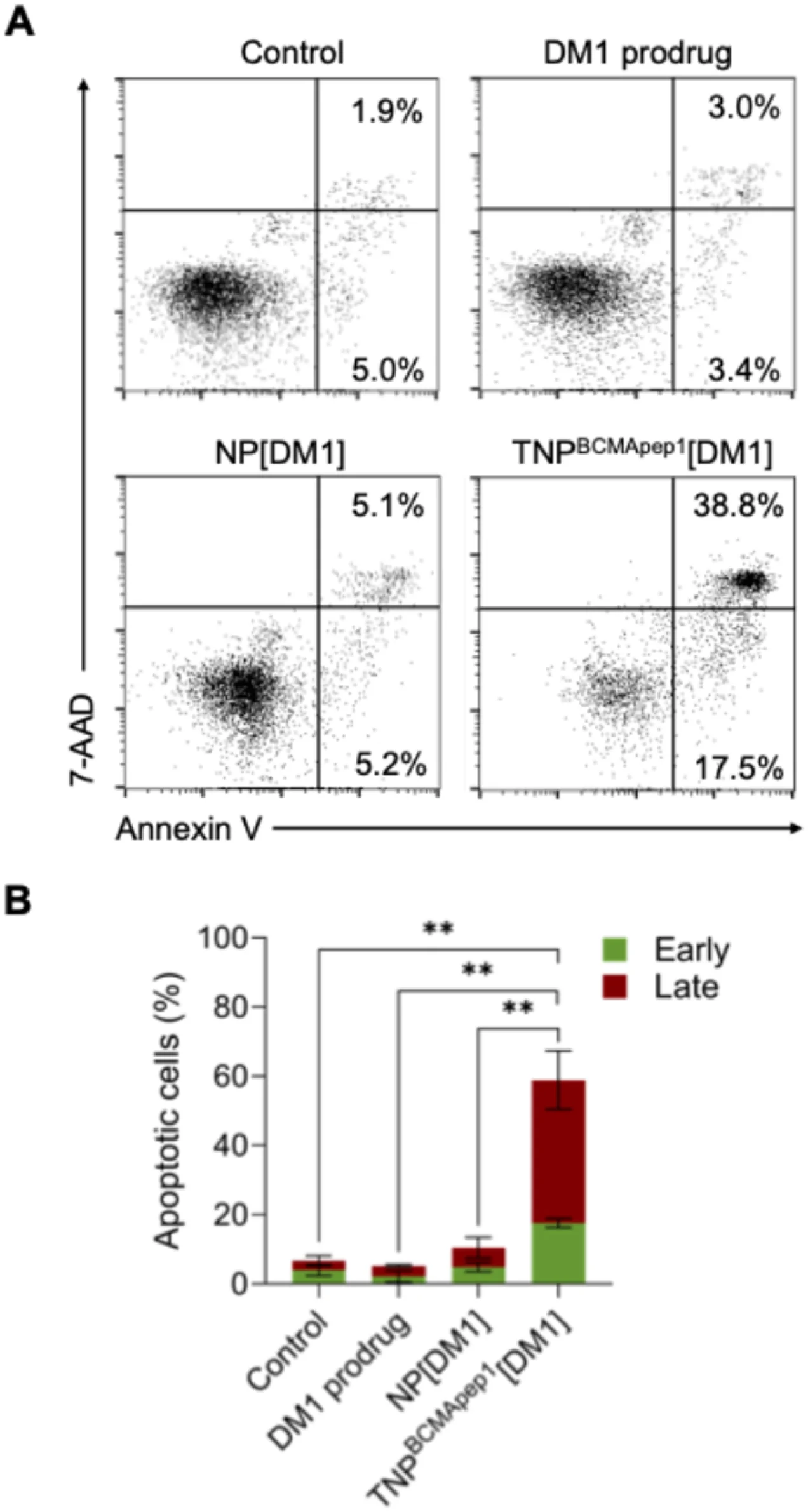
Evaluation of apoptotic cell death following treatment with DM1 prodrug-loaded liposomal nanoparticles. (A) Analysis of apoptosis using Annexin V-Alexa Fluor 488/7-amino-actinomycin D (7-AAD) staining and flow cytometry. NCI-H929 cells were treated with 500 nM DM1-equivalent doses of free DM1 prodrug, NP[DM1], or TNP^BCMApep1^[DM1] for 3 h, washed with PBS, and then incubated in a fresh medium for the remaining time to complete 48 h-incubation. (B) Quantification of early and late apoptotic cells from panel (A). TNP^BCMApep1^[DM1] was prepared with 0.5% BCMApep1 density. All experiments were performed in triplicate, and data represent mean ± SD. Statistical significance was assessed using an unpaired *t*-test. **, *P* < 0.01.

A notable finding in our study is that TNP^BCMApep1^[DM1] outperformed NP[DM1], highlighting its potential to overcome key challenges associated with the off-target toxicity of ADCs in MM therapy by selectively targeting MM cells. In 2020, belantamab madofotin (belamaf), a representative ADC composed of a humanized BCMA monovalent antibody conjugated to a tubulin polymerization inhibitor monomethyl auristatin F (MMAF), received accelerated FDA approval for the relapsed and/or refractory MM. However, a subsequent phase III failed to demonstrate a significant progression free survival (PSF) benefit compared to standard of care, leading to the withdrawal of its conditional approval in 2023.^33,34^ Additionally, in clinical studies, the use of belamaf has revealed severe ocular toxicities, largely attributed to MMAF that causes off-target damage to the corneal epithelial cells.^35^ As shown by belamaf, ADCs face persistent challenges, including off-target toxicity, formulation stability, and a narrow therapeutic index.^36^ Although previous studies have explored BCMA-targeted nanoparticles by conjugating anti-BCMA antibodies to the surface of nanoparticles, these particles with high affinity ligands are most likely to encounter similar limitations to conventional ADCs.^37–39^ In our study, we addressed these challenges by loading a prodrug form of tubulin inhibitor DM1 into NP and TNP^BCMApep1^ formulations. This approach allows the formulation and maintenance of the stability of the payload with the stable linkage of the DM1 prodrug until it reaches the target MM cells, thereby minimizing premature release and systemic toxicity.^28^ Importantly, TNP^BCMApep1^[DM1] demonstrated enhanced apoptotic activity, indicating improved therapeutic efficacy over NP[DM1]. In addition, our findings suggest that the incorporation of the BCMA-targeting ligand on the nanoparticle surface may not only enhance MM cell selectivity and the therapeutic index but also reduce off-target side effects commonly associated with ADC-based strategies.

## Conclusions

In contrast to conventional high-affinity targeted drug delivery approaches, the peptide-targeted nanoparticles described here selectively target MM cells, which is achieved through tuning avidity by optimizing the peptide valency for selectivity over high binding. Properly tuned targeted nanoparticles can effectively distinguish between MM and non-MM cells. Specifically, we identified BCMApep1, a peptide with weak affinity, as an optimal candidate for BCMA targeting through our evaluations, following confirmation of its specificity through competitive inhibition assays. Nanoparticles functionalized with BCMApep1 exhibited preferential uptake by MM cells, highlighting their potential in cancer cell-selective drug delivery. Additionally, DM1 prodrugs were successfully loaded into TNP^BCMApep1^, enabling stable and selective delivery into MM cells. These findings suggest significant advantages over traditional therapies, particularly in reducing off-target toxicity and improving formulation stability.

In future studies, we plan to extend this research to include *in vivo* biodistribution analyses to assess particle accumulation at tumor sites and evaluate potential systemic off-target effects. Comparative studies incorporating previously developed MM-targeting peptides into similar nanoparticle formulations could further elucidate the mechanisms governing tumor cell uptake and accumulation. Additionally, assessing the *in vivo* therapeutic efficacy of TNP^BCMApep1^[DM1] will be necessary for clinical translation. Overall, TNP^BCMApep1^[DM1] holds significant promise as a next-generation therapeutic strategy, offering a safer and more effective approach for selective drug delivery in the treatment of multiple myeloma.

## Author contributions

Gyoyeon Hwang: conceptualization, resources, investigation, validation, visualization, methodology, writing – original draft. Raeleen H. Sobetski: investigation, resources, validation. Doga E. Karakus: investigation. Emily K. Bromley: investigation. Basar Bilgicer: conceptualization, methodology, resources, supervision, funding acquisition, writing – review & editing.

## Conflicts of interest

There are no conflicts to declare.

## Supplementary Material

NA-OLF-D6NA00398B-s001

## Data Availability

The authors confirm that the data supporting the findings of this study are available within the article and in the supplementary information (SI). Supplementary information is available. See DOI: https://doi.org/10.1039/d6na00398b.
