# Supplementary material for: Identification of B-cell maturation antigen (BCMA)-targeting peptides and selective drug delivery to multiple myeloma using peptide-targeted DM1 prodrug-loaded nanoparticles
Source: Nanoscale Adv. 2026 Oct 9. Online ahead of print. doi: 10.1039/d6na00398b (PMC13726103; doi:10.1039/d6na00398b)
Supplement: Supplementary file 1 [file NA-OLF-D6NA00398B-s001.pdf]

Supplementary Information (SI) for Nanoscale Advances.
This journal is © The Royal Society of Chemistry

Supplementary Information (SI)

Identification of B-cell maturation antigen (BCMA)-targeting peptides and selective drug delivery to multiple myeloma using peptide-targeted DM1 prodrug-loaded nanoparticles

Gyoyeon Hwang[1,3,4], Raeleen H. Sobetski[1,3,4], Doga E. Karakus[2], Emily K. Bromley[1,3,4], Basar Bilgicer[1,2,3,4,*]

[1] Department of Chemical and Biomolecular Engineering, University of Notre Dame, Notre Dame, IN, USA

[2] Department of Chemistry and Biochemistry, University of Notre Dame, Notre Dame, IN, USA

[3] Mike and Josie Harper Cancer Research Institute, University of Notre Dame, Notre Dame, IN, USA

[4] Berthiaume Institute for Precision Health, University of Notre Dame, Notre Dame, IN, USA

*Corresponding author: Basar Bilgicer; Email: bbilgicer@nd.edu

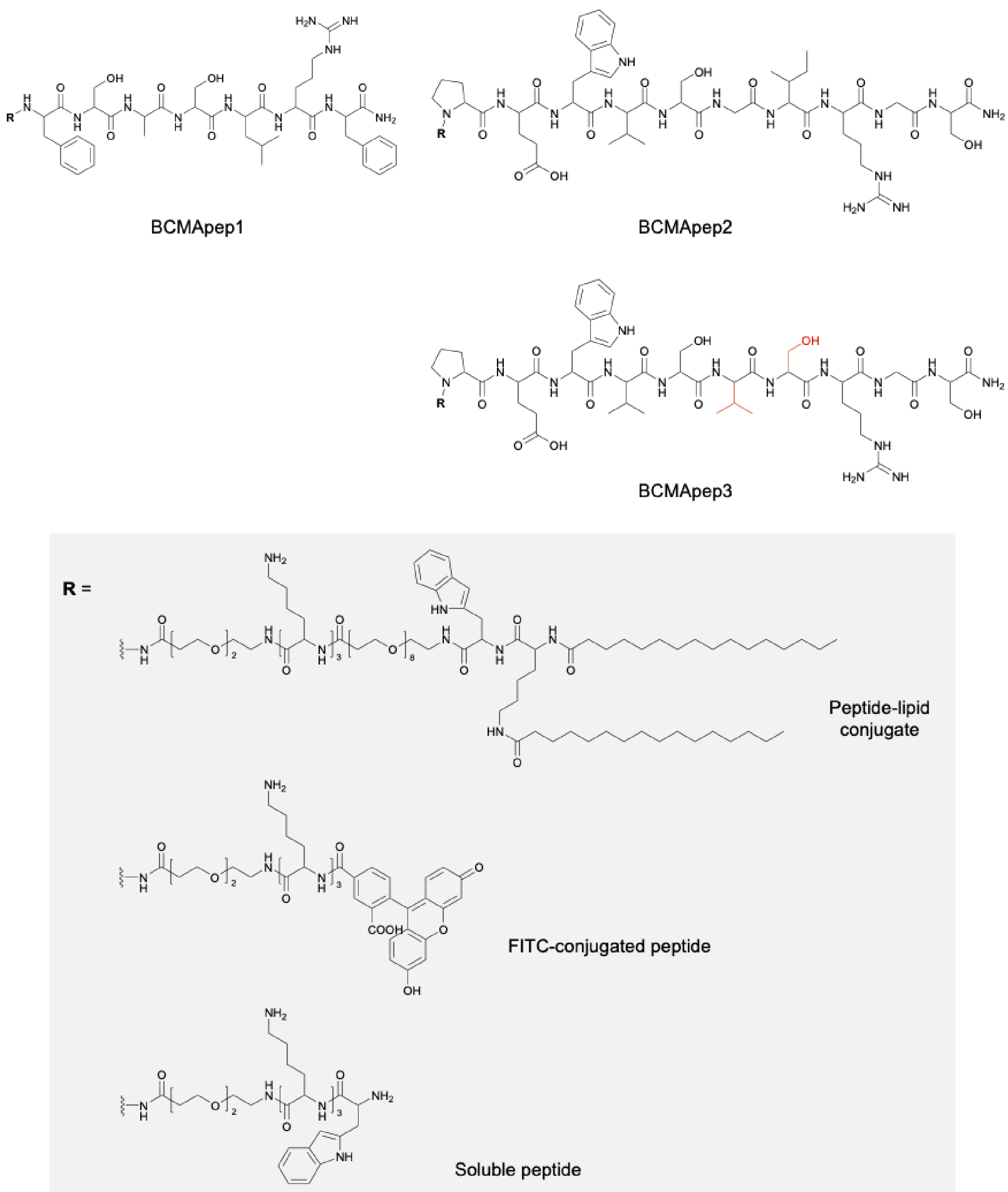

Fig. S1 Chemical structures of BCMApep-lipid conjugates, FITC-conjugated BCMApep, and soluble BCMApep. The R group on BCMApep1, BCMApep2, and BCMApep3 indicates a carbon moiety that connects the rest of the molecule in peptide-lipid conjugates, FITC-conjugated peptides, or soluble peptide forms. Colored (red) side chains are modified amino acids in BCMApep3 that were modified from BCMApep2.

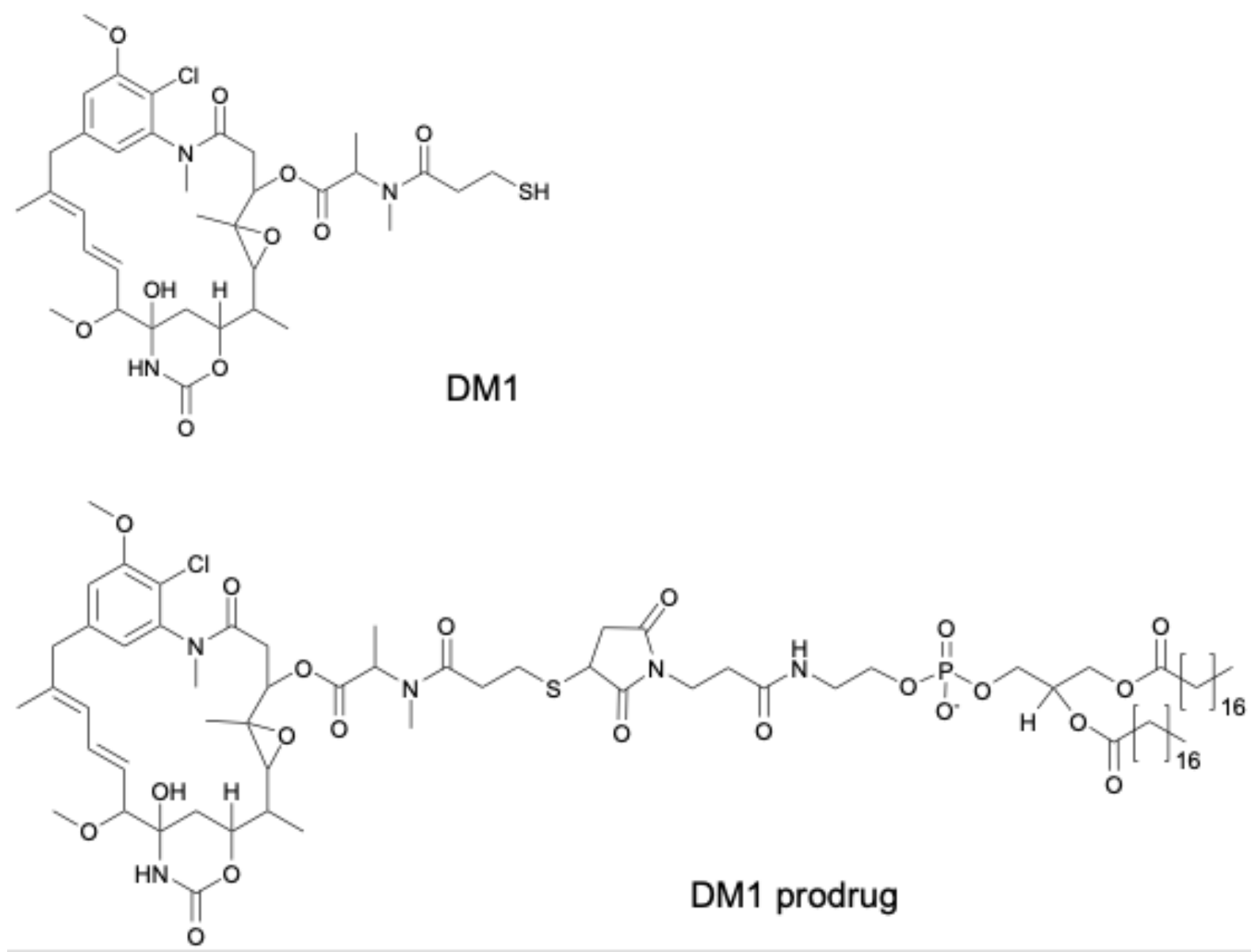

Fig. S2 Chemical structures of DM1 and DM1 prodrug.

Table S1. Mass spectrometry data for the synthesized molecules in this study.

Molecule	Expected mass (Da)	Observed mass (Da)
FITC-conjugated BCMApep1	1726.9	1727.0
FITC-conjugated BCMApep2	1987.0	1988.0
FITC-conjugated BCMApep3	2003.0	2004.0
BCMApep1-lipid conjugate	2582.7	2583.8
BCMApep2-lipid conjugate	2842.8	2843.9
BCMApep3-lipid conjugate	2858.8	2859.9
Soluble BCMApep1	1554.9	1555.9
DM1 prodrug	1634.8	1659.0 (+Na adduct)

Table S2. Particle sizes of the liposomal nanoparticles.[a]

	Non-drug loaded particles		DM1 prodrug loaded particles	
Peptide density (%)	Effective diameter (nm)	Polydispersity	Effective diameter (nm)	Polydispersity
0	72.4 ± 1.0<br>NP	0.06 ± 0.02	75.1 ± 1.0<br>NP[DM1]	0.06 ± 0.03
TNP^{BCMApep1}				
0.1	71.7 ± 0.5	0.04 ± 0.03		
0.2			75.9 ± 0.8	0.07 ± 0.04
0.25	71.9 ± 1.1	0.03 ± 0.04		
0.5	72.3 ± 0.6	0.04 ± 0.03	76.6 ± 0.7<br>TNP^{BMCApep1}[DM1]	0.07 ± 0.03
0.75	72.7 ± 0.9	0.09 ± 0.04		
1	73.1 ± 1.0	0.14 ± 0.04	82.4 ± 1.1	0.14 ± 0.04
2	75.9 ± 2.2	0.17 ± 0.11		
TNP^{BCMApep2}				
0.1	73.2 ± 1.0	0.09 ± 0.05		
0.25	74.4 ± 0.6	0.07 ± 0.06		
0.5	71.5 ± 1.3	0.03 ± 0.03		
0.75	70.5 ± 0.3	0.05 ± 0.02		
1	73.8 ± 0.6	0.06 ± 0.04		
TNP^{BCMApep3}				
0.1	71.0 ± 1.9	0.06 ± 0.03		
0.25	70.5 ± 1.3	0.05 ± 0.02		
0.5	67.4 ± 1.2	0.08 ± 0.01		
0.75	67.3 ± 1.5	0.08 ± 0.05		
1	67.9 ± 0.8	0.08 ± 0.03		

[a] The mean particle size was determined based on intensity (%) using dynamic light scattering measurements. Data represent mean ± SD (n = 5).

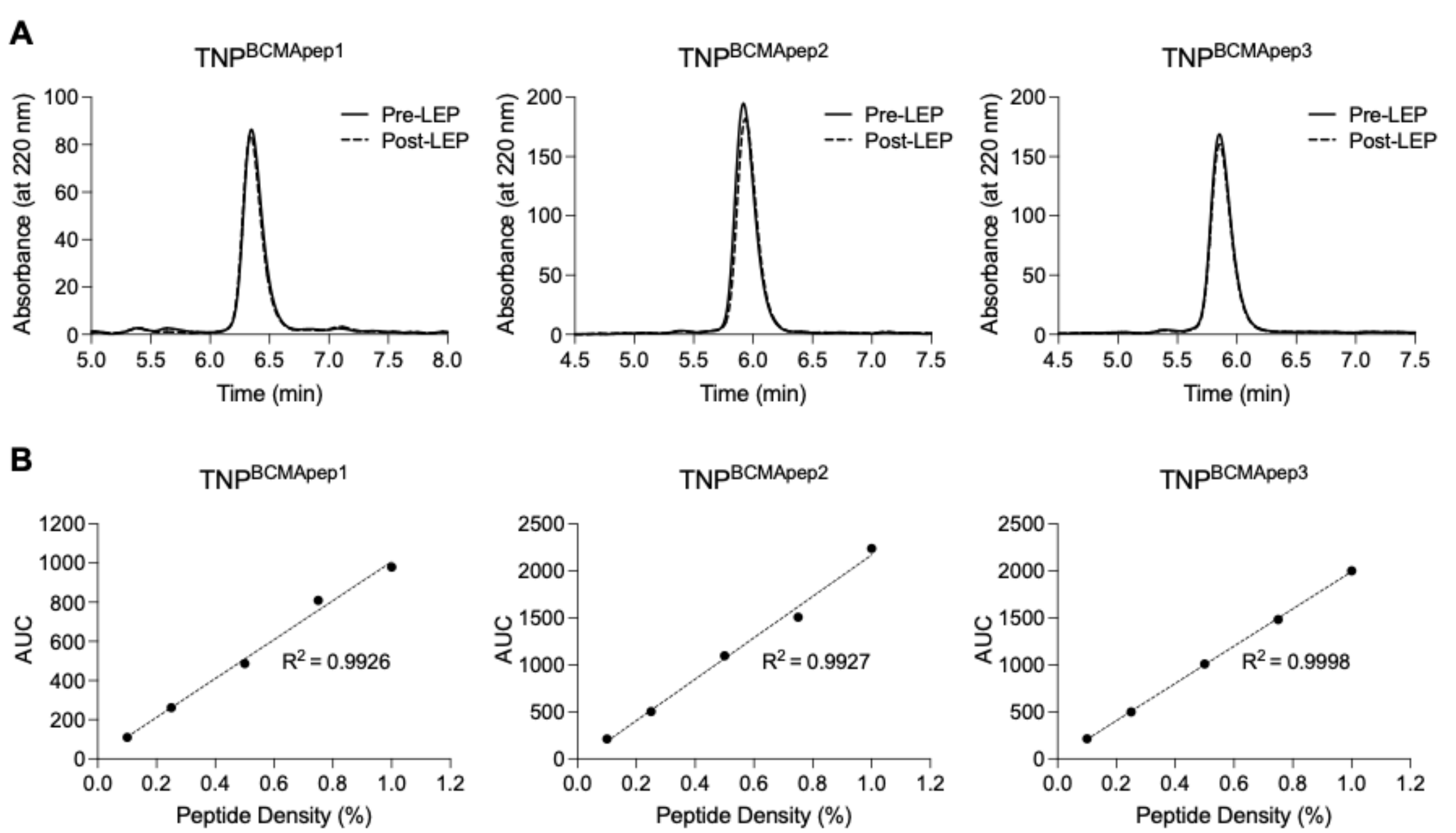

Fig. S3 Loading efficiency of peptides onto $TNP^{BCMApep}$. Liposomes were extruded and subjected to liposome extruder purification (LEP) using a 30 nm pore-size membrane. Both pre-LEP and post-LEP liposomes were then analyzed using RP-HPLC. (A) RP-HPLC chromatograms demonstrating the 220 nm absorbance peaks of peptide-lipid conjugates in both pre-LEP and post-LEP liposomes. Each peptide-lipid conjugate was loaded in liposomes at a 1% peptide density. (B) The area under the curve (AUC) of the 220 nm absorbance peak for peptide-lipid conjugates in post-LEP $TNP^{BCMApep}$ samples at varying peptide densities (0.1%, 0.25%, 0.5%, 0.75%, and 1%), as determined by RP-HPLC.

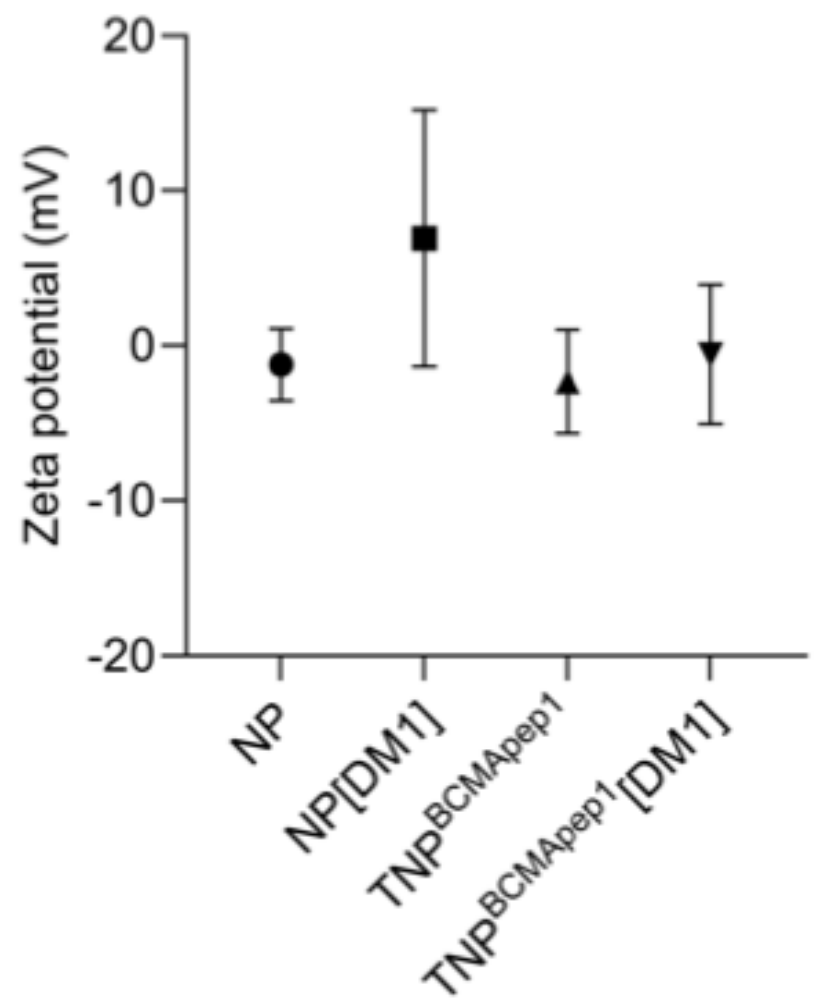

Fig. S4 Zeta potential analysis of NP, NP[DM1], $\text{TNP}^{\text{BCMApep1}}$, and $\text{TNP}^{\text{BCMApep1}}$[DM1]. $\text{TNP}^{\text{BCMApep1}}$, and $\text{TNP}^{\text{BCMApep1}}$[DM1] were prepared with 0.5% BCMApep1. The zeta potential values of NP, NP[DM1], $\text{TNP}^{\text{BCMApep1}}$, and $\text{TNP}^{\text{BCMApep1}}$[DM1] were -1.2 ± 2.3, 6.9 ± 8.3, -2.3 ± 3.3, and -0.6 ± 4.5, respectively. Data represent mean ± SD (n = 5).

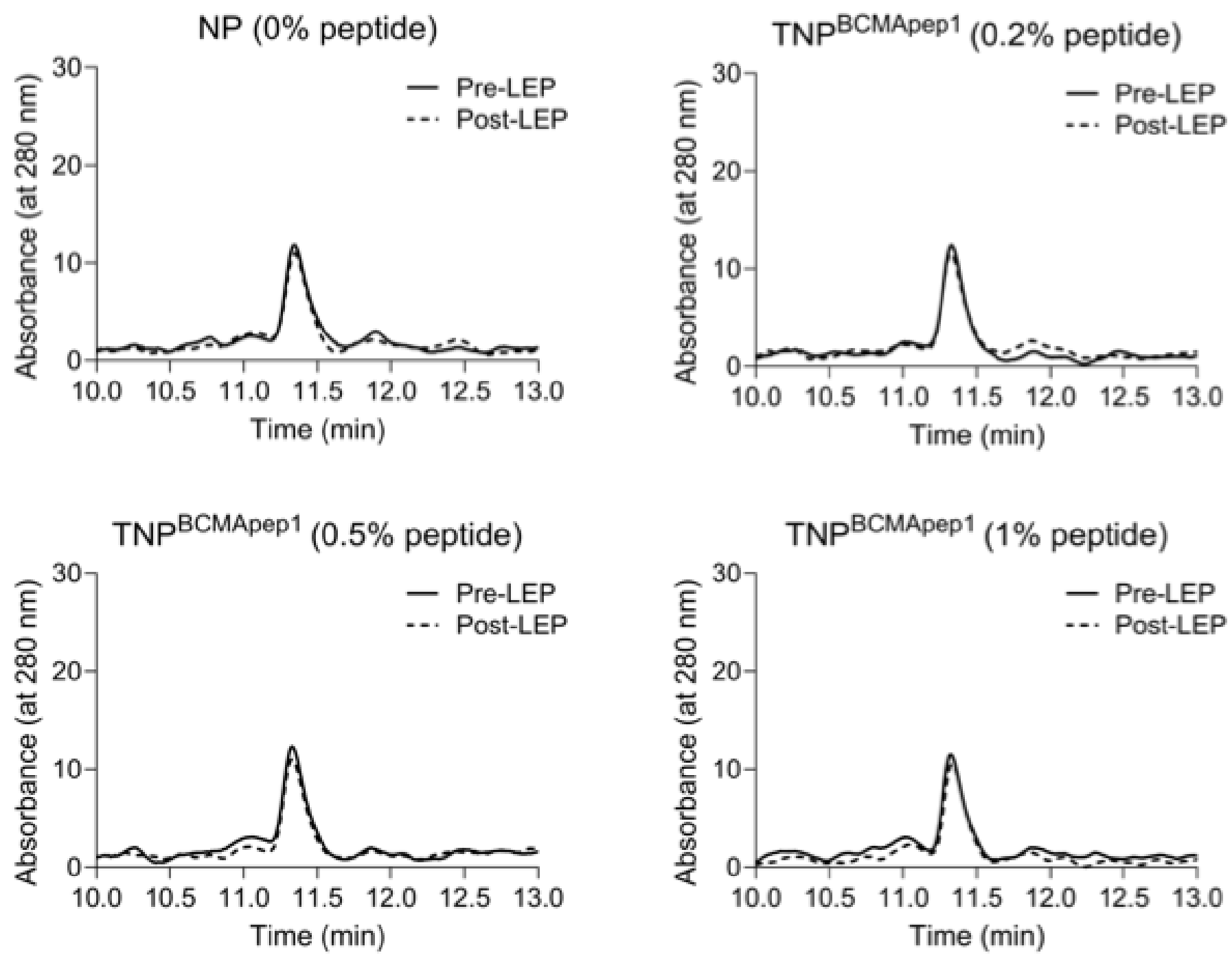

Fig. S5 Loading efficiency of DM1 prodrug onto NP[DM1] (0% BCMApep1 density) and $\text{TNP}^{\text{BCMApep1}}$[DM1] at varying BCMApep1 densities (0.2%, 0.5%, and 1%). Particles were formulated with DM1 prodrug at a loading of 5 mol%. Liposomes were extruded and subjected to liposome extruder purification (LEP) using a 30 nm pore-size membrane. Both pre-LEP and post-LEP liposomes were then analyzed using RP-HPLC. RP-HPLC chromatograms demonstrating the 280 nm absorbance peaks of DM1 prodrug in both pre-LEP and post-LEP liposomes. Loading efficiencies were >96% for all formulations.

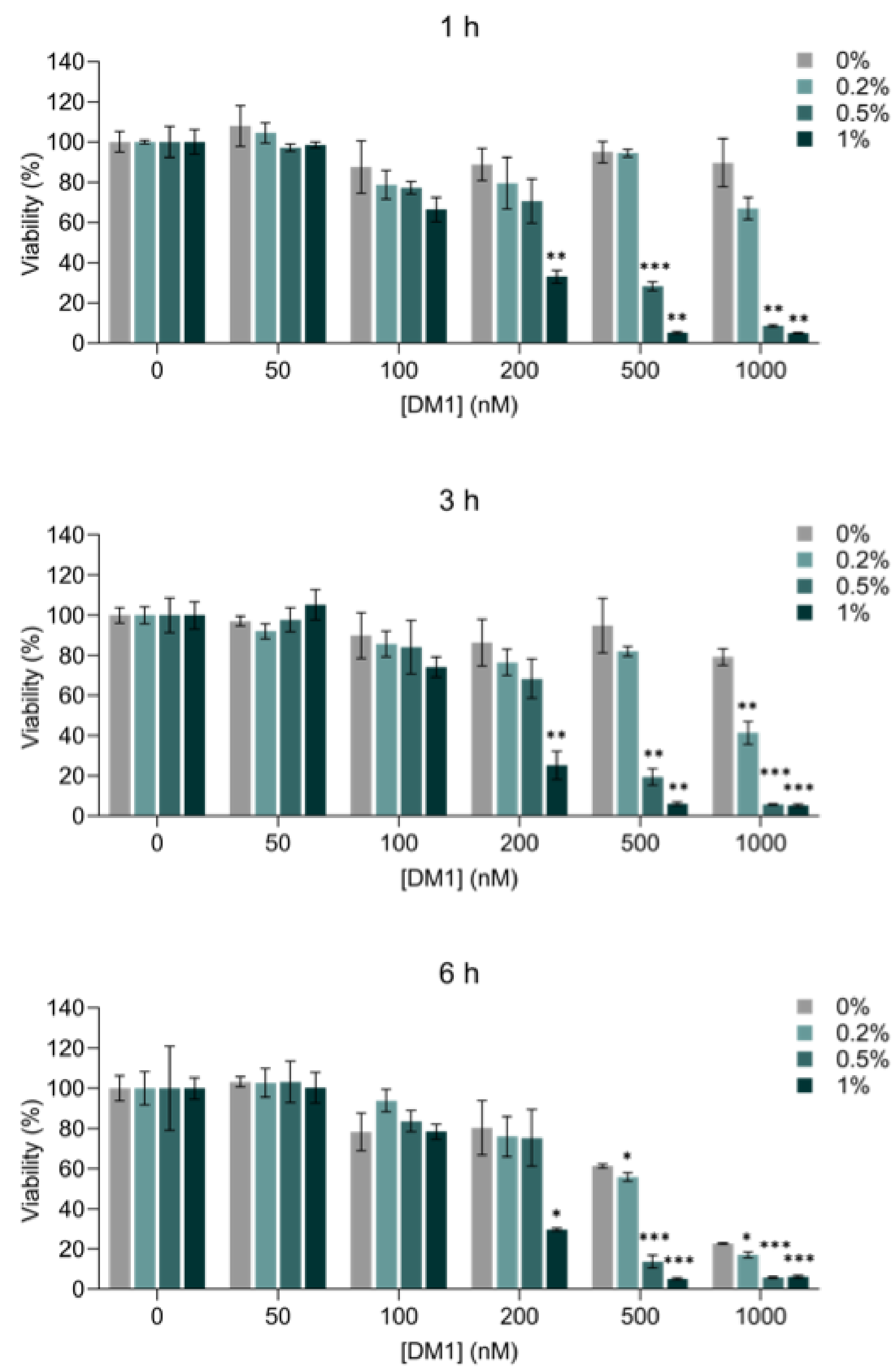

Fig. S6 Pulse dosing approach in NCI-H929 cells using NP[DM1] (0% BCMApep1 density) and $TNP^{BCMApep1}$[DM1] at varying BCMApep1 densities (0.2%, 0.5%, and 1%) shown in the Fig. 5B. Cells were incubated with each formulation for 1, 3, or 6 h, washed with PBS, and then maintained in fresh media for the remaining time to complete a 48 h-incubation. All experiments were performed in triplicates, and data represent mean ± SD. Statistical significance represents comparison of viability between NP[DM1] (0% BCMApep1 density) and $TNP^{BCMApep1}$[DM1] at varying BCMApep1 densities (0.2%, 0.5%, or 1%). *P*-values were calculated using an unpaired t-test. ***, $P<0.001$; **, $P<0.01$; *, $P<0.05$

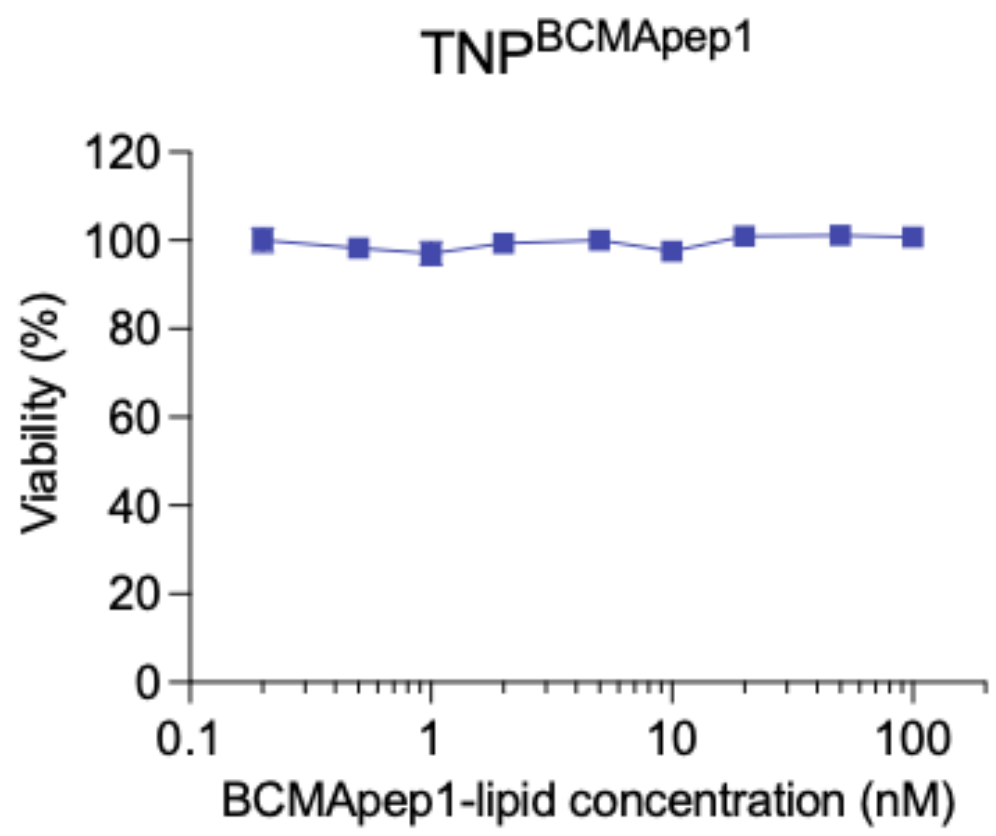

Fig. S7 Cytotoxicity of TNP^{BCMApep1}. NCI-H929 cells were incubated with TNP^{BCMApep1} (without DM1 prodrug loading) for 48 h. TNP^{BCMApep1} were prepared with 0.5% BCMApep1 density. All experiments were performed in triplicates, and data represent mean ± SD.
